# A radiographic criteria checklist to determine reasons for errors, resulting in sub-optimal routine shoulder projections

**DOI:** 10.4102/hsag.v24i0.1038

**Published:** 2019-08-14

**Authors:** Ida-Keshia Sebelego, Belinda van der Merwe, Jeanette du Plessis

**Affiliations:** 1 Department of Clinical Sciences, Central University of Technology, Bloemfontein, South Africa

**Keywords:** routine shoulder projections, radiographic criteria, AP external rotation, lateral-Y projections, anatomical structures, radiographic technique

## Abstract

**Background:**

Optimal shoulder images must adhere to specific radiographic criteria before they are sent to the radiologist for reporting. Repeat x-rays of the shoulder may increase radiation exposure to the patient.

**Aim:**

The aims of this study were to determine whether images adhered to the required radiographic criteria for routine shoulder imaging and to identify possible reasons for non-adherence.

**Setting:**

The study was conducted at an imaging department at a tertiary academic hospital in Bloemfontein, South Africa.

**Methods:**

A criteria checklist compiled from literature was used to evaluate 578 routine shoulder images including anteroposterior (AP) with external rotation and lateral-Y (LAT-Y) projections. The checklist determined whether the shoulder images adhered to the criteria with regard to the anatomy included, positioning and technical factors, such as inclusion of the correct anatomical lead marker. Data were analysed using SAS Version 9.2 statistical software.

**Results:**

More than 80% of the AP external rotation images included unnecessary anatomical structures owing to incorrect centring. In four out of seven criteria pertaining to positioning for AP external rotation imaging, at least 70% of images were performed incorrectly. Four-sided collimation was not present in more than 50% of both AP external rotation and LAT-Y images because of incorrect centring, while more than 30% of shoulder images presented with anatomical digital markers.

**Conclusion:**

The application of criteria required for shoulder imaging must be addressed at the participating imaging department to improve overall patient care. An in-service training session is recommended to enhance the radiographic technique with regard to routine shoulder projections.

## Introduction

The quality of radiographic images plays an integral role in the diagnosis and subsequent care of patients (Hobbs 2007). Therefore, patient positioning has to be correct, the region of interest (ROI) needs to be included (Brown 2013) and the necessary radiation safety precautions must be applied.

With x-ray projections of the shoulder, the most sensitive regional organs that may receive scatter ionising radiation are the thyroid gland and the breast. Radiographers are responsible for patients with regard to radiation protection during radiographic imaging (Bontrager & Lampignano 2014). According to the European Commission (s.d.), international standards pertaining to radiation protection are based on justification, optimisation and dose limitation. Repeating x-ray projections owing to positioning error, wrong use of technical factors, poor communication with the patient and improper collimation contribute to unnecessary exposure to radiation that could have been avoided by the radiographer (Bontrager & Lampignano 2014; Bushong 2008).

Collimation influences the diagnostic quality of the radiographic image. Collimation minimises scatter radiation to improve the visibility of the recorded detail (in either conventional or digital imaging), reduces the dose to the patient (Bontrager & Lampignano 2014; Herrmann et al. 2012; McQuillen Martensen 2015; Uffmann & Schaefer-Prokop 2009) and reduces the histogram analysis errors associated with digital imaging (McQuillen Martensen 2015). If an x-ray image does not present with four-sided collimation borders, it implies that the primary beam was not restricted and/or an incorrect centring point was used (Bontrager & Lampignano 2014; McQuillen Martensen 2015).

An x-ray image with an anatomical lead marker may serve as legal documentation in a court of law, and by implication, not placing the anatomical lead marker on a cassette or imaging plate (IP) before exposure may have medico-legal implications (Image Gently s.d.; McQuillen Martensen 2011). Radiologists may refuse to report on an x-ray image that has no anatomical lead marker placed prior to exposure (Platt & Strudwick 2009; Titley & Cosson 2014) or does not have an anatomical lead marker, and such images may have to be repeated. Radiographers registered with the Health Professions Council of South Africa (HPCSA) must have the patient’s best interest at heart (HPCSA 2008). Failure to place anatomical lead markers prior to an x-ray exposure is an indication of not acting in the patient’s best interest (Platt & Strudwick 2009). Radiographic images without anatomical lead markers may result in projections being repeated, and consequently lead to an increase in radiation exposure to the patient.

## Aims and objectives

Although the goal of imaging departments should be to deliver high-quality x-ray images, the lead researcher observed that in clinical practice it is often challenging for radiographers to obtain routine x-ray projections of the shoulder that adhere to the established radiographic criteria, which lead to the study being conducted. The aims of this study were to determine to what extent routine x-ray projections of the shoulder adhered to specific radiographic criteria and to identify possible reasons for images of the shoulder not adhering to these criteria. The objectives were to (1) benchmark from literature the radiographic criteria for routine anteroposterior (AP) projection (external rotation) and lateral-Y (LAT-Y) projection of the shoulder and (2) compile the radiographic criteria checklist based on the literature review to identify causes contributing to images failing to meet the requirements.

## Methods

### Design

The study was approached from the pragmatic paradigm focussing on problem-solving. This paradigm was chosen because of the problem observed, and specific methodologies were used to provide solutions to address the problem at hand (Hall 2013). A descriptive, evaluative and explanatory study was conducted. The study was conducted at an imaging department at a tertiary academic hospital in Bloemfontein, South Africa. To achieve the aims, AP external rotation and LAT-Y shoulder projections were assessed with regard to anatomical structures included, radiographic positioning, collimation and the use of anatomical lead markers. The images of routine shoulder projections were evaluated using a radiographic criteria checklist (Appendix 1) to identify criteria that radiographers had not considered during assessment of the routine projections of the shoulder before sending images for reporting. Data were collected using a radiographic criteria checklist to determine whether routine shoulder images that were sent through for reporting, adhered to the required radiographic criteria. The checklist made provision for open-ended comments to allow the researcher to explore possible reasons for a specific shoulder image not adhering to the required radiographic criteria.

### Research instrument

The checklist used in this study consisted of radiographic criteria used to formulate an ‘opinion or judgment about a particular practice’; in other words, to retrospectively critique the routine shoulder images acquired by the radiographers at the participating imaging department. By selecting ‘yes’ or ‘no’, the researcher could determine if the shoulder projections adhered to the requirements as stipulated in the checklist (Delport & Roestenburg 2011; Leedy & Ormrod 2005). The checklist was based on various publications regarding radiographic criteria to evaluate the two routine projections of the shoulder (Ballinger & Frank 1999; Bontrager & Lampinano 2014; Greathouse 1998; McQuillen Martensen 2011, 2015).

The content validity of the checklist was pilot-tested by six participants, which included two lecturers from a higher education institution, three radiologists and an orthopaedic surgeon who is a shoulder specialist. The participants were selected based on their easy accessibility/availability to the researcher and were excluded from the main study. No changes were made to the checklist after pilot testing.

The checklist (Appendix 1) was divided into three main sections, namely the anatomical structures included in the projection, positioning criteria and technical factors. The section on anatomical structures referred to the important anatomy required to be included on the image for a specific shoulder examination (Table 1). Three anatomical criteria for the AP external rotation projection were required, namely (a) the superior scapula; (b) two-thirds of the clavicle; and (c) one-third of the proximal humerus (Ballinger & Frank 1999; Bontrager & Lampinano 2014; Greathouse 1998; McQuillen Martensen 2011, 2015). Five anatomical criteria for the LAT-Y projection included (a) the superior and inferior angles of the scapula; (b) the glenohumeral (GH) joint; (c) the proximal humerus; (d) the coracoid process; and (e) the acromion process (Ballinger & Frank 1999; Bontrager & Lampinano 2014; Greathouse 1998; McQuillen Martensen 2011, 2015). Table 1 outlines the positioning criteria for the routine shoulder projections (Ballinger & Frank 1990; Bontrager & Lampinano 2014; Greathouse 1998; McQuillen Martensen 2011, 2015).

**TABLE 1:** Positioning criteria for routine shoulder projections (Ballinger & Frank 1999; Bontrager & Lampinano 2014; Greathouse 1998; McQuillen Martensen 2011, 2015).

AP (external rotation) projection	LAT-Y projection
No visible motion on the image	No visible motion on the image
Greater tubercle in profile (on lateral aspects of proximal humerus)	Acromion, coracoid processes and scapular body form a Y (true lateral)
Lesser tubercle positioned between the greater tubercle and the humeral head (lesser tubercle superimposing the humeral head)	Scapula not magnified
No superimposition of the superolateral border of the scapula over the ribs	Acromion projected lateral
Humeral head slightly overlaps the glenoid cavity	Coracoid processes superimpose the clavicle or projected below the clavicle
Humeral head is in profile	Lateral and vertebral border of the scapula is superimposed
Humerus is aligned parallel with the body	Humeral head superimposes the base of the Y
Clavicle demonstrated horizontally	Relationship between the humeral head and glenoid cavity is seen clearly
Superior scapula angle is superimposed by the midclavicle	Scapular body seen on end without superimposition of ribs
Glenohumeral joint and coracoid process are in the centre of the collimation	Shaft of humerus superimpose body of scapula
-	Shaft of the humerus not superimposed by the ribs
-	Mid-scapular body/humeral head and surgical neck are at the centre of the image

AP, anteroposterior; LAT-Y, lateral-Y.

The section on technical factors identified two important aspects, namely: (a) visibility of the correct anatomical lead marker and (b) collimation. When the correct centring point was used and collimation applied prior to exposure, clear four-sided collimation borders were visible on the AP external rotation and LAT-Y images (Bontrager & Lampinano 2014; McQuillen Martensen 2015).

### Sample selection

A simple random sampling technique was used to select images of routine shoulder projections for evaluation, thus preventing bias and ensuring that all routine shoulder projections had an equal chance of inclusion in the research (Goddard & Melville 2001; Strydom 2011). All images of routine shoulder projections, irrespective of having been performed by a student, supplementary, community service or qualified radiographer, were included for evaluation. This culminated in a total of 578 shoulder x-ray examinations performed on 578 patients during the period 10 August 2015 to 30 January 2016. Images of the shoulder that did not include the AP (external rotation) and LAT-Y projections were excluded.

### Data collection procedure

Raw/static images were evaluated on display monitors because the researcher could undo any post-processing of the shoulder images obtained by the radiographers prior to evaluation. Considering that images are automatically deleted on the display monitors after a certain time to ensure continuous space available in the digital storage system, evaluation of the shoulder images was performed thrice a week during the study period. Images of routine shoulder projections on the Picture Archiving and Communication System (PACS) were not evaluated because radiographers commonly use post-processing tools to ‘fix’ the images, such as altering collimation before sending the images to the PACS.

The shoulder images that were evaluated included 578 AP external rotation and 578 LAT-Y projections. Every time the researcher collected data at the participating imaging department, a note was made of the display monitor used to search for routine shoulder projections, the patient’s name and the date of the examination. The notes made were for the researcher’s own use to assist her to keep track of data collection equipment used, data collection start and end points.

### Data analysis

The data from the hard copy checklist were captured electronically in two Microsoft Excel spreadsheets for the AP external rotation projection and the LAT-Y projection, respectively. Additional comments were documented and all comments showing similarities to reason why images did not adhere to the criteria were used to formulate sub-sections (Table 3) to facilitate further statistical analysis of data.

Data analysis was done using the SAS Version 9.2 statistical analysis software (SAS Institute Inc., Cary, NC). Descriptive statistics, namely frequencies and percentages, were calculated for categorical data. Means and standard deviations or medians and percentiles were calculated for numerical data.

### Ethical considerations

The researcher obtained ethical approval (ECUFS 100/2015) from the Research Ethics Committee of the Faculty of Health Sciences, University of the Free State, the Department of Health in the Free State, the head of Clinical Services and the director of the participating imaging department. No patients were directly involved in the study; therefore, informed consent was not required. No names of the patients and the participating imaging department, where the data had been collected, were mentioned or used in the study, which ensured anonymity and confidentiality. All information collected was managed in a strictly ethical and confidential manner.

## Results

The results are presented in accordance with the anatomical structures that must be included for the AP (external rotation) and LAT-Y projection, positioning criteria and technical considerations that must be considered during positioning and the most evident reasons for non-adherence of radiographic criteria requirements. For each criterion used, the analysis will be first for the AP (external rotation) projection and then the LAT-Y projection of the shoulder.

### Anatomical structures included

#### Anteroposterior external rotation projection of the shoulder

**FIGURE 1:**
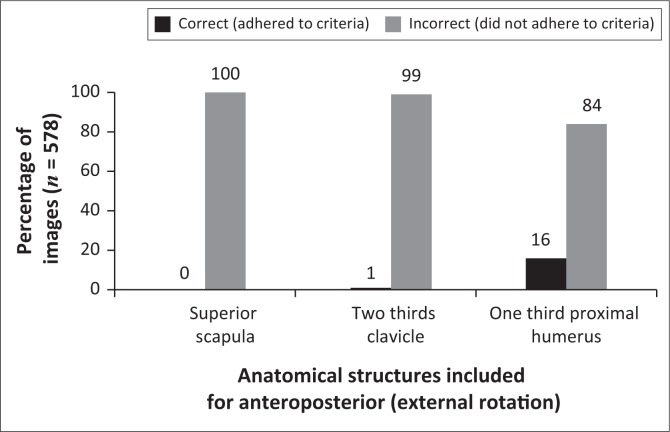
Specific anatomical structures included for an anteroposterior (external rotation) shoulder projections.

Figure 1 shows a comparison between correct and incorrect anatomical structures included for the AP external rotation projection. All of the images (100%) demonstrated anatomical structures inferior to the superior angle of the scapula (which did not adhere to the criteria), while on 99% of images, more than two-thirds of the clavicle were observed (which did not adhere to the criteria). Results showed that 84% of the images included more than one-third of the proximal humerus, whereas it should only include one-third of the proximal humerus in the collimation field (Table 1).

#### Lateral-Y projection of the shoulder

**FIGURE 2:**
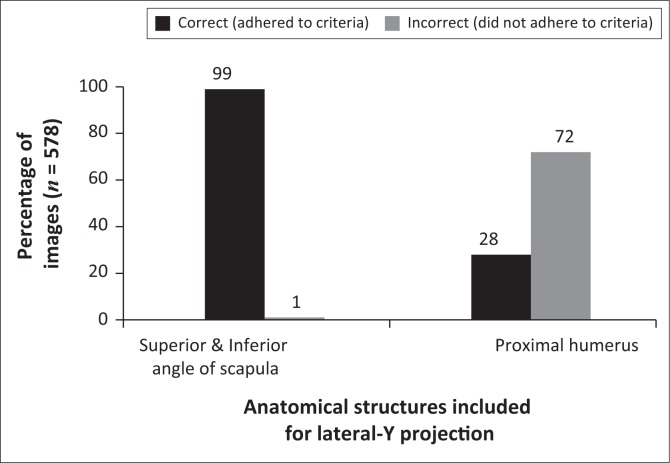
Specific anatomical structures included for a lateral-Y shoulder projection.

Figure 2 shows that only 1% of the LAT-Y shoulder projections were performed incorrectly with regard to the superior and inferior angles of the scapula. However, 72% of the images did not meet the criteria as they included additional anatomical structures, whereas they should have included the proximal humerus only.

### Positioning criteria

#### Anteroposterior external rotation projection of the shoulder

**FIGURE 3:**
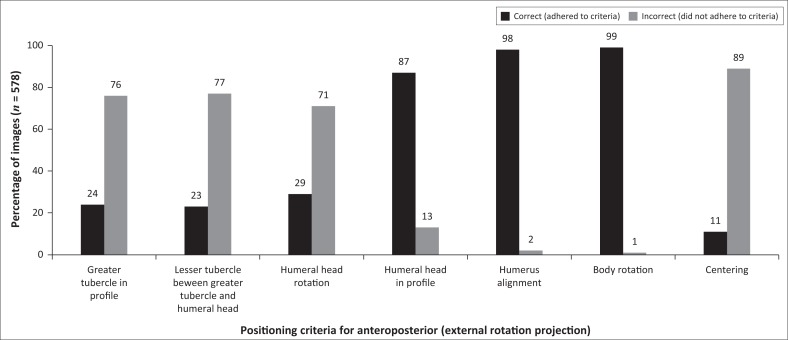
Criteria for positioning of the anteroposterior projection (external rotation) of the shoulder.

The AP external rotation projection has to adhere to specific criteria in relation to positioning (Table 1). Figure 3 shows that 89% of the images did not adhere to the correct centring, with the glenohumeral joint and coracoid process being in the centre of the collimation field. More than 70% of the images showed incorrect humeral head rotation (humeral head should slightly overlap the glenoid cavity). Furthermore, 77% of the images did not demonstrate the correct lesser tubercle (LT) rotation. Correct LT rotation was performed when the LT was positioned between the greater tubercle (GT) and humeral head, whereas correct GT rotation demonstrated the GT in profile laterally to the proximal humerus (Table 1). As shown in Figure 3, 76% of the images evaluated did not adhere to the criterion ‘greater tubercle in profile’.

#### Lateral-Y projection of the shoulder

**FIGURE 4:**
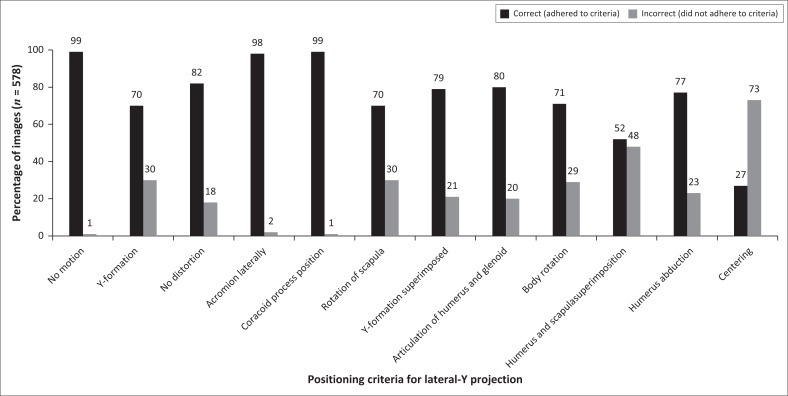
Criteria for positioning of the lateral-Y projection of the shoulder.

Specific criteria for positioning are required for LAT-Y projections (Table 1). Figure 4 indicates four main issues of concern: centring, humerus and scapula superimposition, rotation of and Y-formation of the scapula. The correct centring for a LAT-Y shoulder projection is achieved when the mid-scapular body or the humeral head and surgical neck are centred in the collimation field. Figure 4 shows that 73% of images did not illustrate correct centring. Forty-eight per cent of LAT-Y shoulder images did not demonstrate the shaft of the humerus superimposing over the scapular body. Y-formation refers to the acromion, coracoid process and scapular body forming a ‘Y’, which was not observed in 30% of images, implying that rotation of the scapula (lateral and vertebral border of scapula superimposed) was incorrect on these images.

### Technical considerations

Four-sided collimation is visible when the primary beam is limited and correct centring has been applied to include only the anatomical structures of interest. Table 2 shows that more than 50% of routine shoulder projections, both AP and LAT-Y, did not demonstrate four-sided collimation. Furthermore, the anatomical lead marker was not visible on 34% of the AP external rotation and 39% of the LAT-Y projections.

**TABLE 2:** Technical considerations: collimation and anatomical lead marker placement.

Technical aspect	Percentage of images (*n* = 578)	
	Correct	Incorrect
**Four-sided collimation visible on routine shoulder images**		
AP (external rotation)	47	53
LAT-Y	48	52
**Anatomical lead marker placement in collimation prior to exposure**		
AP (external rotation)	66	34
LAT-Y	61	39

AP, anteroposterior; LAT-Y, lateral-Y.

### Most evident reasons for non-adherence

The lead researcher added comments in the open-ended sections of the checklist when routine shoulder images did not adhere to the radiographic criteria requirements. Various reasons for non-adherence were identified. Table 3 summarises the most evident reasons for non-adherence to the criteria for AP (external rotation) and LAT-Y shoulder images.

**TABLE 3:** Most evident reasons for non-adherence to radiographic criteria required for shoulder imaging (*n* = 578).

Variable	Reasons for non-adherence to criteria	Percentage
**AP (external rotation)**	**Anatomical structures included**	
	Whole scapula inclusion	100
	Whole clavicle inclusion	99
	Two-thirds of humerus included	77
	**Positioning criteria**	
	Fracture and dislocations present	14
	Centred too inferiorly	24
	Centred inferiorly and medially	16
	Inferior angle of the scapula at centre	4
	Middle of lateral border of the scapula at centre	8
	Middle of vertebral border at centre	4
	Middle of scapular body at centre of the image	10
	**Technical considerations**	
	Collimation applied superiorly and inferiorly	4
	One-sided anatomy, third cervical vertebrae (C3) to sacrum	27
	One-sided anatomy, seventh cervical vertebrae (C7) to tenth thoracic vertebrae (T10)	33
	Digital anatomical lead marker used	33
**LAT-Y**	**Anatomical structures included**	
	Inferior angle of scapula excluded	1
	Whole humerus inclusion	12
	Two-thirds of humerus included	60
	**Positioning criteria**	
	Fracture and dislocations present	14
	Centred too inferiorly	10
	Centred medially	9
	Inferior angle of scapula at centre	5
	Rib cage at centre	5
	Shaft of humerus at centre of the image	4
	Foreshortened scapula	17
	Under-rotation of scapula	22
	**Technical considerations**	
	Collimation applied, medially, laterally and superiorly	6
	One-sided anatomy, third cervical vertebrae (C3) to sacrum	15
	One-sided anatomy, seventh cervical vertebrae (C7) to tenth thoracic vertebrae (T10)	42
	Digital anatomical lead marker used	38

AP, anteroposterior; LAT-Y, lateral-Y.

## Discussion

Figure 1 illustrates that the radiographers included more than the necessary anatomy during imaging of the AP external rotation shoulder projection. In the researchers’ opinion, radiographers probably are not familiar with the anatomical structures that must be included for an AP external rotation projection, or possibly might have been taught to include the whole scapula for AP shoulder projections. The whole scapula was included for all AP external rotation images and 99% of images included the whole clavicle (Table 3). During the pilot study, two pilot participants (radiologist and orthopaedic surgeon) who worked at the participating imaging department recommended that the whole scapula be included for AP external rotation projections, indicating a possible reason why the radiographers at the participating imaging department include the whole scapula during imaging of the AP external rotation projection.

A notable finding was that most of the LAT-Y shoulder projections showed inclusion of the correct anatomical structures, with exception of the proximal humerus. As shown in Figure 2, 72% of the projections were incorrect because either two-thirds of the proximal humerus or the whole humerus were included in the collimation field. Twelve per cent of the images included two-thirds of the proximal humerus, whereas 60% of images included the whole humerus (Table 3). The researchers assert that the radiographers centred too inferiorly (Table 3 and Figure 4), consequently including more of the proximal humerus in the collimation field.

The results depicted in Figure 3 show that four positioning criteria were problematic during imaging of the AP external rotation projection. More than 70% of each of these four criteria were incorrect, namely centring, humeral head rotation (humeral head should slightly overlap the glenoid cavity), which resulted in the GT (greater tubercle) and the LT (lesser tubercle) not being demonstrated optimally. Optimal humeral head rotation was not achieved owing to several cases presenting with shoulder dislocations and fractures (14%), as indicated in Table 3. Thus, the GT was not positioned at the lateral aspect of the proximal humerus, and the LT was not positioned between the GT and humeral head. It is acceptable practice that when a patient presents with fractures, no arm rotation is performed. This is because a fracture can damage the arteries and nerves situated at the shoulder joint (Bontrager & Lampignano 2014) when the arm is moved.

Overall, it appears as if the radiographers did not rotate the patient’s arm externally to demonstrate the GT in profile, and the LT between the GT and humeral head. Furthermore, 89% of AP external rotation projections demonstrated incorrect centring. The radiographers either centred too inferiorly or too medially to the correct centring point. Consequently, structures such as the inferior angle and middle of the scapula were at the centre of the collimation field instead of the GH joint and coracoid process (Table 3). Hence, more than the necessary anatomical structures were included in the collimation field owing to an incorrect centring point (Figure 1).

The LAT-Y shoulder projections adhered to more than 70% of the documented positioning criteria, except for humerus and scapula superimposition and centring (Figure 4). Less than 30% of the LAT-Y shoulder projections did not adhere to the criteria requirements for various reasons, the most prominent criteria detected during data collection being under-rotation of the scapula (22%), shoulder dislocations (14%) and the foreshortening of the scapula (17%). Even when patients present with shoulder dislocations or have experienced acute trauma, a true lateral of the scapula can still be obtained because no movement of the arm is required, only body rotation (Goud et al. 2008; Sanders & Jersey 2005).

The relationship between the humeral head and the glenoid cavity (articulation of humeral head and glenoid) will not be visible on a LAT-Y shoulder projection if the patient presents with a dislocation (American Academy of Orthopedic Surgeons/AAOS 2010; Quillen, Wuchner & Hatch 2004). Therefore, 20% of the LAT-Y shoulder projections did not demonstrate articulation of the humeral head and glenoid, which could be attributed to shoulder dislocations (Figure 2 and Table 3). Humerus and scapula superimposition was not achieved in 48% of LAT-Y shoulder projections owing to 14% of images presenting with fractures and dislocations, and therefore the humerus superimposed the chest cavity (ribs) of the patient. Anatomical structures either inferior or medial to the correct centring point were at the centre of the collimation field (Table 3). These incorrect anatomical structures included the inferior angle of the scapula (5%), the shaft of the humerus (4%) and the ribcage (5%).

Unnecessary inclusion of anatomical structures reflects a lack of adherence to the ALARA principle (as low as reasonably achievable) when considering radiation exposure during imaging. Radiographers must demonstrate optimal collimation practices at all times that contribute to the ALARA principle and provide optimal x-ray images (Uffmann & Schaefer-Prokop 2009). More than 50% of the routine shoulder projections did not demonstrate four-sided collimation for both AP external rotation and LAT-Y shoulder projections (Table 2).

When the researcher had undone all post-processing performed by radiographers, such as collimation (refer to ‘Data collection procedure’ section), in 4% of AP external rotation shoulder projections, collimation was applied only superior and inferior to the ROI (Table 3). However, in 6% of LAT-Y shoulder projections, collimation was only applied medial and lateral to the ROI. The anatomical structures included in both routine shoulder projections most commonly were one-sided anatomy that included third cervical vertebrae (C3) to sacrum, or one-sided anatomy that included seventh cervical vertebrae (C7) to tenth thoracic vertebrae (T10), owing to incorrect centring and not applying collimation effectively (Table 3). One-sided anatomy refers to the side of importance (left or right) that was determined from the spinous process of the vertebral column to the lateral end of the clavicle or humerus on the side of interest. Because of ineffective collimation practices, sensitive organs, such as the thyroid gland, breast and gonads, were exposed to radiation unnecessarily.

The results on the use of anatomical lead markers showed that the radiographers employed anatomical lead markers for more than 60% of routine shoulder projections (Table 2). It was found that 34% of the AP external rotation projections and 39% of the LAT-Y projections presented with digital/electronic anatomical markers. Table 3 shows that 33% of AP external rotation and 38% LAT-Y images had digital anatomical markers, indicating that the radiographers placed a digital anatomical marker after an exposure was made, which in this case is not regarded as good practice as it can be challenged in a court of law and may pose medico-legal consequences (Image Gently s.d.; McQuillen Martensen 2011).

The non-adherence to the radiographic criteria of the shoulder images could be because of the level of training of the radiographers. The images were obtained by student (second-year Bachelor students, second- and third-year Diploma students), community service, supplementary and qualified radiographers (refer to ‘Sample selection’ section) that have different levels of training. Moreover, the experience of the radiographers in imaging of the shoulder among the aforementioned radiographers also differs. Some were exposed more readily than others to imaging of the shoulder and could learn to adjust their radiographic technique in obtaining optimal images of the shoulder in different cases.

## Conclusion

Student, qualified, community service and supplementary radiographers are compelled to provide quality care to all patients and have the best interest of the patient at heart, as outlined by the South African Health Professions Act No. 56 of 1974 (South African Government 1974). Hence, radiographers must ensure that routine AP external rotation and LAT-Y shoulder projections are performed optimally for diagnosis of shoulder pathology. In this study, evaluation of routine shoulder images confirmed a lack of adherence to the radiographic criteria, such as incorrect centring, ineffective collimation practices, not using anatomical lead markers on the images of the shoulder and the exposure of unnecessary anatomical structures to radiation. Furthermore, factors that could have contributed to non-adherence to the radiographic criteria, such as fractures and dislocations, were also pointed out.

A limitation of the study was that the lead researcher was the only person who evaluated the 578 routine shoulder images, which potentially could have led to errors in the evaluation of the images. Another limitation was that the lead researcher did not consult the diagnostic reports of the radiologists to determine whether the quality of the routine shoulder images prevented them from making a diagnosis or not. However, it is important to note that the checklist was used to determine the reasons for non-adherence to the radiographic image evaluation criteria. Images of trauma patients were included in the study which contributed to the high percentage of non-adherence to the radiographic criteria, specifically in relation to humeral head rotation in demonstrating the GT and the LT optimally for the AP external rotation projection. Therefore, the inclusion of trauma patients in the study was a limitation. The researcher may have been too rigid in her approach with regard to the pilot study and the inclusion of anatomical structures for the AP projection (external rotation). Two participants (radiologist and orthopaedic surgeon) as part of the pilot testing phase indicated that the whole scapula should be included for the AP external rotation projection, as the participating imaging department is exposed to trauma cases and does not want to miss underlying pathologies. However, the checklist was not amended accordingly by the researcher; thus, it was a limitation in the study.

Nevertheless, based on the findings of the study, the lead researcher has a responsibility to recommend an in-service training session at the participating imaging department for the improvement of radiographic technique with regard to routine shoulder projections at the participating imaging department. The researchers strongly recommend that a pre- and post-intervention research study with an in-service training session as intervention on the topic at hand be executed in another study, to determine if such a session would improve radiographers’ practice regarding routine shoulder imaging. The radiographic criteria checklist can also be used by other imaging departments to determine if the images obtained adhere to the specific requirements.

## Appendix 1

### RADIOGRAPHIC CRITERIA CHECKLIST FOR THE ROUTINE AP PROJECTION OF THE SHOULDER (EXTER NAL ROTATION)

**Checklist unique number:**

**Table:**

*CRITERIA 1: ANATOMICAL STRUCTURES INCLUDED IN THE PROJECTION*				For office use only:
	YES	NO	COMMENT	
1.1. Superior scapula				
1.2. 2/3 of clavicle				
1.3. 1/3 Proximal humerus				
***CRITERIA 2: POSITIONING FACTORS***				
2.1. No visible motion on projection				
2.2. Greater tubercle in profile (on lateral aspects of proximal humerus)				
2.3. Lesser tubercle positioned between the greater tubercle and the humeral head (lesser tubercle superimposing the humeral head)				
2.4. No superimposition of superolateral border of scapula over ribs				
2.5. Humeral head slightly overlap glenoid cavity				
2.6. Humeral head is in profile				
2.7. Humerus aligned parallel with the body				
2.8. Clavicle demonstrated horizontally				
2.9. Superior scapula angle superimposed by midclavicle				
2.10. Glenohumeral joint and coracoid process in centre of collimation				
***CRITERIA 3: TECHNICAL FACTORS***				**For office use only:**
3.1. Identification visible				
3.2. Lead marker is visible				
3.3. No artefacts visible				
3.4. Four-sided collimation margins visible before post-processing				
***CRITERIA 4: EXPOSURE FACTORS***				
	**YES**	**NO**	**COMMENT**	
4.1. Bony trabecular detail sharply defined				
4.2. Cortical outlines of the shoulder demonstrated sharply				
4.3. Soft-tissue seen around proximal humerus				
4.4. Average exposure factors (70–80 kvp 16–25 mAs)				
4.5. Exposure index (EI) for shoulder imaging is Non-bucky = 345–689, Bucky = 145–344 (Philips & Agfa)				
4.6. Amount of repeats				
***5. Additional comments:***				
				
				
				
				
				

### RADIOGRAPHIC CRITERIA CHECKLIST FOR THE ROUTINE LATERAL PROJECTION OF THE SHOULDER (Y-VIEW)

**Checklist unique number:**

**Table:**

*CRITERIA 1: ANATOMICAL STRUCTURES INCLUDED IN THE PROJECTION*				For office use only:
	YES	NO	COMMENT	
1.1. Superior and inferior angle of the scapula				
1.2. Glenohumeral joint				
1.3. Proximal humerus				
1.4. Coracoid processes				
1.5. Acromion processes				
***CRITERIA 2: POSITIONING FACTORS***				
2.1. No motion visible on projection x-ray				
2.2. Acromion, coracoid processes and scapular body form a Y (true lateral)				
2.3. Scapula not magnified				
2.4. Acromion projected lateral				
2.5. Coracoid processes superimpose the clavicle or projected below the clavicle				
2.6. Lateral and vertebral border of scapula is superimposed				
2.7. Humeral head superimpose the base of the Y				
2.8. Relationship between the humeral head and glenoid cavity is seen clearly				
2.9. Scapular body seen on end without superimposition of ribs				
2.10. Shaft of humerus superimpose body of scapula				
2.11. Shaft of the humerus not superimposed by ribs				
2.12. Mid-scapular body/ humeral head and surgical neck is at centre of the projection				
***CRITERIA 3: TECHNICAL FACTORS***				**For office use only:**
	**YES**	**NO**	**COMMENT**	
3.1. Identification visible				
3.2. Lead marker is visible				
3.3. No artefacts visible				
3.4. Four collimation margins visible before post-processing				
***CRITERIA 4: EXPOSURE FACTORS***				
4.1. Bony trabecular detail is sharply defined				
4.2. Cortical outlines of the shoulder are sharply demonstrated				
4.3. Soft-tissue seen around shoulder (Lateral and superior region of the shoulder)				
4.4. Average exposure factors (70-80 kvp 16-25 mAs)				
4.5. Exposure index (EI) of for shoulder imaging Non-bucky = 345–689, Bucky = 145–344 (Philips & Agfa)				
4.6. Amount of repeats				
***5. Additional comments:***				
				
				
				
				
				
